# Systematic Analysis and Identification of Stress-Responsive Genes of the NAC Gene Family in *Brachypodium distachyon*

**DOI:** 10.1371/journal.pone.0122027

**Published:** 2015-03-27

**Authors:** Jun You, Lihua Zhang, Bo Song, Xiaoquan Qi, Zhulong Chan

**Affiliations:** 1 Key Laboratory of Plant Germplasm Enhancement and Specialty Agriculture, Wuhan Botanical Garden, Chinese Academy of Sciences, Wuhan, China; 2 Key Laboratory of Plant Molecular Physiology, Institute of Botany, Chinese Academy of Sciences, Beijing, China; Key Laboratory of Horticultural Plant Biology (MOE), CHINA

## Abstract

Plant-specific NAC proteins are one of the largest families of transcription factors in plants, and members of this family have been characterized with roles in the regulation of diverse biological processes, including development and stress responses. In the present study, we identified 101 putative NAC domain-encoding genes (*BdNACs*) through systematic sequence analysis in *Brachypodium distachyon*, a new model plant of family Poaceae. BdNAC proteins were phylogenetically clustered into 13 groups, and each group possesses similar motif compositions. Phylogenetic analysis using known stress-related NACs from Arabidopsis and rice as query sequences identified 18 *BdNACs* as putative stress-responsive genes. *In silico* promoter analysis showed that almost all *BdNAC* genes contain putative stress-related cis-elements in their promoter regions. Expression profile of *BdNAC* genes in response to abiotic stresses and phytohormones was analyzed by quantitative real-time RT-PCR. Several putative stress-responsive *BdNAC* genes, including *BdNAC003* and *BdNAC044* which is ortholog of known stress-responsive rice gene *SNAC1* and *SNAC2*, respectively, were highly regulated by multiple abiotic stresses and stress-related phytohormone treatments. Taken together, our results presented here would be helpful in laying the foundation for understanding of the complex mechanisms of NAC mediated abiotic stress signaling transduction pathways in *B*. *distachyon*.

## Introduction

Plants growth and productivity are frequently threatened by various environmental stresses for their sessile nature. To cope with these stresses, plants have evolved a range of physiological and biochemical responses [1] and a complex of signaling transduction pathways [2, 3]. Transcription factors (TFs) are one of the critical regulatory proteins involved in abiotic stress responses and play important roles downstream of stress signaling cascades. TFs regulate the expression of a subset of stress-related genes and modulate the plant resistance to environmental stresses. Members of DREB, MYB, MYC, bZIP, zinc-finger and NAC families have been well characterized with roles in the regulation of plant stress responses [3–5].

NAC TFs comprise a plant-specific gene family, which is characterized by highly conserved NAC domains. NAC was derived from the names of the three firstly described proteins containing the similar conserved DNA-binding domain, namely NAM (no apical meristem), ATAF1/2 and CUC2 (cup-shaped cotyledon) [6]. Numerous members of this family were identified in the sequenced plant species, including 105 genes in Arabidopsis [7], 140 genes in rice [8], 152 genes in soybean [9] and 163 genes in poplar [10]. The conserved NAC domain is approximately 160 amino acids in length and contains five conserved regions (A to E) [7, 11]. The crystal structure of the DNA-binding NAC domain of Arabidopsis ANAC019 and rice SNAC1 shows that NAC domain contains a unique TF fold consisting of a twisted beta-sheet [12, 13]. At least five different DNA-binding sites for the NAC TFs have been identified in Arabidopsis, including the drought-responsive NAC recognition sequence (NACRS) containing CATGT [14]. The C-terminal parts of NAC proteins are highly diverse, and function as transcriptional activator or repressor [6, 15]. A common feature of NAC protein C-terminal regions is the frequent occurrence of simple amino acid repeats and regions rich in serine and threonine, proline and glutamine, or acidic residues [6]. Interestingly, both the NAC domain and C-terminal region possess the capacity for mediating protein-protein interactions [15, 16].

Although over 100 members of NAC family have been identified in many plant species [7–10], only a few of them have been functionally characterized to date. The originally reported NAC proteins are involved in various aspects of plant development. Arabidopsis CUC2 protein plays important role in controlling the formation of boundary cell [17]. *AtNAC1* is induced by auxin and mediates auxin signaling to promote lateral root development [16]. More recently, NAC proteins were found to participate in regulating senescence [18] and formation of secondary walls [19]. NAC proteins were also reported to participate in abiotic and biotic stress responses. Three Arabidopsis NAC proteins ANAC019, ANAC055 and ANAC072 were identified by yeast one-hybrid using promoter region of *ERD1*, and overexpression of either of these genes significantly improved drought resistance in transgenic plants [14]. Recently, Arabidopsis NAC proteins JUB1, NTL4 and VNI2 were documented to participate in stress responses by leaf senescence regulation [18, 20, 21]. Increasing evidence indicated that some NAC TFs play crucial roles in protecting plants against abiotic stresses in rice, such as SNAC1 [22], SNAC2 [23, 24], OsNAC5 [25], and OsNAC10 [26]. *SNAC1* was specifically induced in the guard cells under drought stress condition. Overexpression of *SNAC1* in rice resulted in stomata closure and improved drought resistance in the drought-stressed field condition while the yield of transgenic plants was not affected under normal growth condition [22]. Overexpression of *OsNAC10* or *OsNAC5* driven by a root-specific promoter *RCc3* in rice also increased grain yield under field drought condition [25, 26].

*Brachypodium distachyon* is the first member to be sequenced within the Pooideae subfamily [27], that includes most cool season cereal (such as wheat and barley), forage and turf grasses. Due to its small genome size and plant size, short life cycle, and efficient cultivation and transformation systems, *B*. *distachyon* has become a model system for functional genomics studies in temperate cereals [28]. Valdivia et al. [29] reported eight *SWN* (*SECONDARY WALL NAC*) genes in *B*. *distachyon*, and revealed the function of *BdSWN5* in secondary cell-wall synthesis and programmed cell death. However, genome-wide systematic analysis of *B*. *distachyon* NAC TFs continues to be lacking. In the present study, 101 *NAC* genes were identified from the *B*. *distachyon* Bd21 genome and a detailed evolution, gene structure and conservation domain/motif analyses were performed. Evolutionary relationship of *B*. *distachyon* NAC protein with their counterparts from monocot rice and eudicot Arabidopsis was comprehensively analyzed, and several putative stress-responsive *BdNAC* genes were identified. Furthermore, the expression profiles of *BdNACs* in response to several abiotic stresses and phytohormones were examined using quantitative real-time RT-PCR (qPCR), which provided direct clues for selection of appropriate stress-responsive candidate genes for further functional analyses.

## Materials and Methods

### Identification and annotation of the BdNAC proteins in *B*. *distachyon*

Three different approaches were applied to identify putative NAC domain containing proteins in *B*. *distachyon*. Initially, *B*. *distachyon* annotation database (MIPS/JGI v1.2) at Phytozome v9.1 (http://www.phytozome.net) was searched using the keywords ‘NAC’. Then, sequences of Arabidopsis and rice NAC proteins were downloaded from TAIR release 10 (The Arabidopsis Information Resource, http://www.Arabidopsis.org/) and RGAP release 7 (Rice Genome Annotation Project http://rice.plantbiology.msu.edu/), respectively. BLASTP searches were subsequently performed to identify homologous proteins of Arabidopsis and rice NACs in *B*. *distachyon*. Finally, HMM profiles of the NAM domains (PF02365) in the Pfam database (http://pfam.xfam.org/) were searched against the Phytozome database of *B*. *distachyon*. Similar searches were also performed at NCBI database against the non-redundant protein sequence of *B*. *distachyon* to eliminate possible exclusions of any additional NAC members. All non-redundant sequences were manually checked for the NAM domain and compared with the NAC family in the transcription factor database PlantTFDB (http://planttfdb.cbi.pku.edu.cn/) and GrassTFDB (http://grassius.org/grasstfdb.html). Other conserved domains in addition to NAM domain were also identified in the Pfam database. Transmembrane motifs in the sequences were identified with TMHMM Server v.2.0 (http://www.cbs.dtu.dk/services/TMHMM/) using default parameters.

NAC proteins in other land plants were identified by BLASTP search in Phytozome and plantTFDB using well-known NAC proteins from Arabidopsis, and NCBI database was used for searching the NAC proteins in red and green algae. All putative non-redundant sequences were assessed with Pfam and PROSITE profiling in InterPro database (http://www.ebi.ac.uk/interpro/).

### Chromosomal location, gene structure and duplication analysis for BdNACs

*BdNAC* genes were mapped to the *B*. *distachyon* genome according to their position information from Phytozome. Gene structure display server program (GSDS, http://gsds.cbi.pku.edu.cn/index.php) was utilized to show exon/intron structure of each *BdNAC* gene by comparison of the coding sequences with their corresponding genomic sequences from Phytozome. The duplication pattern for each NAC gene was analyzed using MCScanX software (http://chibba.pgml.uga.edu/mcscan2/) according to the previous description [30]. Briefly, whole-genome BLASTP analysis of *B*. *distachyon* was performed using local blast+ software with e-value under 1e-5, and an-outfmt 6 format output was produced. The blast outputs and position of all protein-coding genes were imported into MCScanX software (http://chibba.pgml.uga.edu/mcscan2/), and genes were classified into various types of duplications including segmental, tandem, proximal and dispersed under a default criterion.

### Sequence alignment, phylogenetic analysis and motif identification

Multiple alignments of the full-length protein sequences were performed with ClustalX (version 1.83). The unrooted phylogenetic trees were constructed with MEGA5 software using the neighbor-joining (NJ) method and the bootstrap test was carried out with 1000 iterations. Pairwise deletion mode was used to ensure that the highly divergent C-terminal domains could contribute to the topology of the NJ tree. To study the phylogenetic relationship of BdNAC proteins along with their counterparts in Arabidopsis and rice, further multiple sequence alignment including BdNACs and NACs from Arabidopsis (ANACs) and rice (ONACs) was performed using ClustalX (version 1.83). However, when the multiple sequence alignment was used for constructing phylogenetic tree, the error "some pairwise distances could not be estimated" was reported by MEGA5 software due to great variation of several NAC pairwises. Therefore, eight NACs from rice (ONAC003, ONAC081, ONAC113, ONAC114, ONAC115, ONAC116, ONAC117, ONAC130) and one Arabidopsis NAC (ANAC088) with great variations were removed, then the unrooted tree was plotted as described above. The conserved motifs were identified using Multiple Expectation Maximization for Motif Elicitation (MEME) program version 4.9.1 (http://meme.nbcr.net/meme/cgi-bin/meme.cgi) with the following parameters: number of repetitions—any, maximum number of motifs—20, and the optimum motif widths were constrained to between 6 and 200 residues. All motifs discovered by MEME were searched in InterPro database (http://www.ebi.ac.uk/interpro/).

### Plant materials, growth conditions and treatments

The community standard diploid inbred line of *B*. *distachyon*, Bd21, was used in this study. To measure transcript levels of the NAC family members in *B*. *distachyon* under various stresses and phytohormone treatment conditions, *B*. *distachyon* plants were grown in a greenhouse controlled at 22°C with a 16 h light/8 h dark cycle. The 3-week-old seedlings were subjected to various abiotic stress and phytohormone treatments. For drought stress, the seedlings were grown without watering and sampled at 15 d after treatment. For salt stress, the seedlings were irrigated with 200 mM NaCl solution and sampled at 6 h after treatment. For cold and heat shock stresses, seedlings were transferred to a growth chamber at 4°C and 42°C, respectively. The above ground tissues were sampled at 6 h after cold stress treatment, and 0.5 h after heat stress treatment. For phytohormone treatments, 3-week-old seedlings were treated with 100 μM abscisic acid (ABA), 300 μMethephon (Eth), 100 μM jasmonic acid (JA) and 100 μM salicylic acid (SA), respectively. The above ground tissues were collected at 6 h after treatments.

### RNA isolation and quantitative real-time RT-PCR

Total RNA was isolated using Plant total RNA Isolation Kit (Sangon, Shanghai, China) according to manufacturer's instructions. Before reverse transcription, total RNA was treated with RNase-free DNase I (Fermentas, Vilnius, Lithuania) for 30 min to degrade possibly contaminated residual genomic DNA. First-strand cDNAs were synthesized from DNaseI-treated total RNA using reverse transcriptase (TOYOBO, Shanghai, China) and oilgo-dT primers according to the manufacturer’s instructions.

Quantitative real-time RT-PCR (qPCR) was performed on a CFX96 Real Time System (Bio-Rad, Hercules, California, USA) using iQ SYBR Green supermix (Bio-Rad) according to the manufacturer’s protocol. The *ubiquitin-conjugating enzyme 18* gene (*UBC18*, *Bradi4g00660*) was used as the endogenous control according to previous study [31]. The relative gene expression levels were determined by the ΔΔCT method as described previously [32]. The qPCR assays were performed with three replicates. The gene-specific primers are listed in S1 Table.

## Results

### Identification of NAC proteins in *Brachypodium distachyon*

The NAC proteins in *B*. *distachyon* were identified by keyword, Hidden Markov Model (HMM) profile and BLAST searches against *B*. *distachyon* annotation (MIPS/JGI v1.2) database at Phytozome v9.1 (http://www.phytozome.net/brachy.php). A total of 69 gene loci with annotations containing the words "NAC" were found by keyword search. BLASTP searches of the predicted *B*. *distachyon* protein database with known NAC proteins from Arabidopsis and rice resulted in 95 non-redundant gene loci, and 48 of them were overlapped with the keyword search result. Above results were subsequence checked by HMM profile of the NAM domain to remove false sequences, and 100 non-redundant gene loci were released. Similar searches were also performed at the NCBI database, and an additional NAC gene (*BdSWN1*, accession number: JQ693422) reported by Valdivia et al. [29] was identified. Search against the Phytozome database indicated the locus ID for *BdSWN1* is Bradi1g76730, and the MIPS v1.2 annotation of Bradi1g76730 is incorrect (S1 Fig.). Taken together, a total of 101 non-redundant gene loci were predicted to encode putative NAC or NAC-like proteins in *B*. *distachyon* (S2 Table).

NAC genes in *B*. *distachyon* are designated as *BdNAC* followed by number 001–101 based on their sequential locations on the chromosomes. Among 101 *BdNAC* genes, 7 (*BdNAC017*, *BdNAC024*, *BdNAC029*, *BdNAC049*, *BdNAC053*, *BdNAC065* and *BdNAC068*) produce alternative spliced transcripts according to the *B*. *distachyon* annotation (MIPS/JGI v1.2) database, and only the splice variants encoding the longest open reading frames were chosen as representatives for subsequent sequence alignments and phylogenetic analyses. Recently, 78,163 high quality expressed sequence tags (ESTs) were obtained [33], and structural gene annotation of 26 *BdNAC* genes was updated (S2 Table). The detailed information of NAC genes in *B*. *distachyon*, including locus ID, chromosome distribution (start sites and end sites) as well as the length of coding sequences was listed in S2 Table.

### Chromosomal distribution and gene duplication of NAC gene family in *B*. *distachyon*

**Fig 1:**
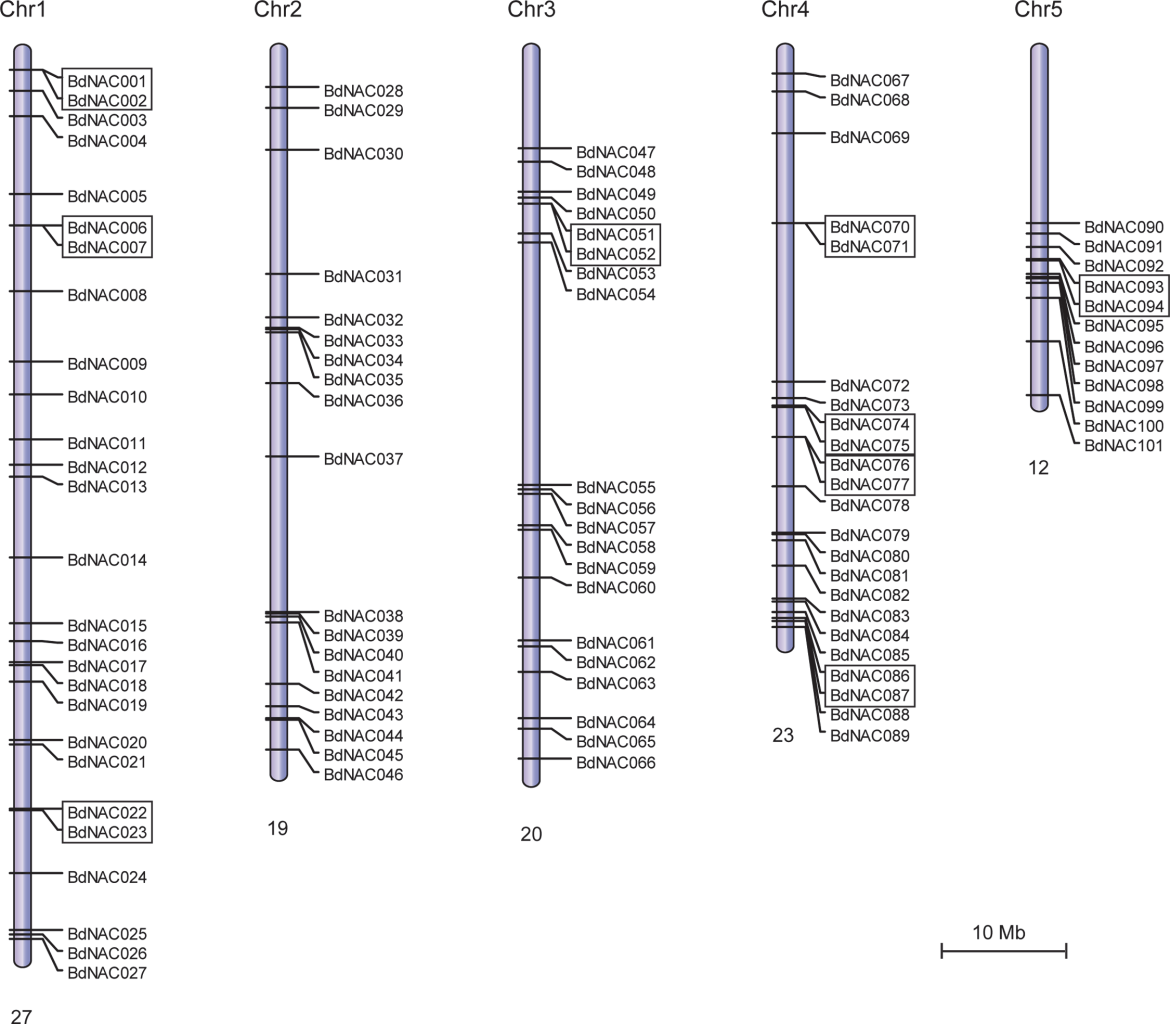
Chromosomal distribution of *NAC* genes in *B*. *distachyon*. Totally 101 *BdNAC* genes were mapped to the 5 chromosomal according to their positions in the *B*. *distachyon* genome. The chromosome number (Chr1–Chr5) was shown on the top of each chromosome. The number below indicated the number of *BdNAC* genes in each chromosome. Nine clusters of *BdNAC* genes were indicated in boxes. The scale bar indicated a chromosomal distance of 10.0 Mb.

The *BdNACs* were mapped to chromosomes based on the coordinates of Phytozome loci. As shown in Fig. 1, 101 *BdNACs* are unevenly distributed on all the 5 chromosomes of *B*. *distachyon*. Chromosome 1 harbored 27 *BdNACs*, the largest number among all chromosomes, whereas, chromosome 5 only contains 12 members. In addition to the number of *BdNACs* distributed on each chromosome, the location of *BdNACs* on each chromosome is also uneven. The *BdNACs* located in chromosomes 4 and 5 are concentrated in the lower end of the arms, and *BdNACs* are non-randomly allocated on other 3 chromosomes. Nine clusters each with 2 *BdNACs* were identified by the criteria that distance between neighboring *BdNACs* is less than 200 kb. Chromosome 4 contains the maximum number (4) of clusters, whereas 3 clusters on chromosome 1, and one each on chromosomes 3 and 5.

**Fig 2:**
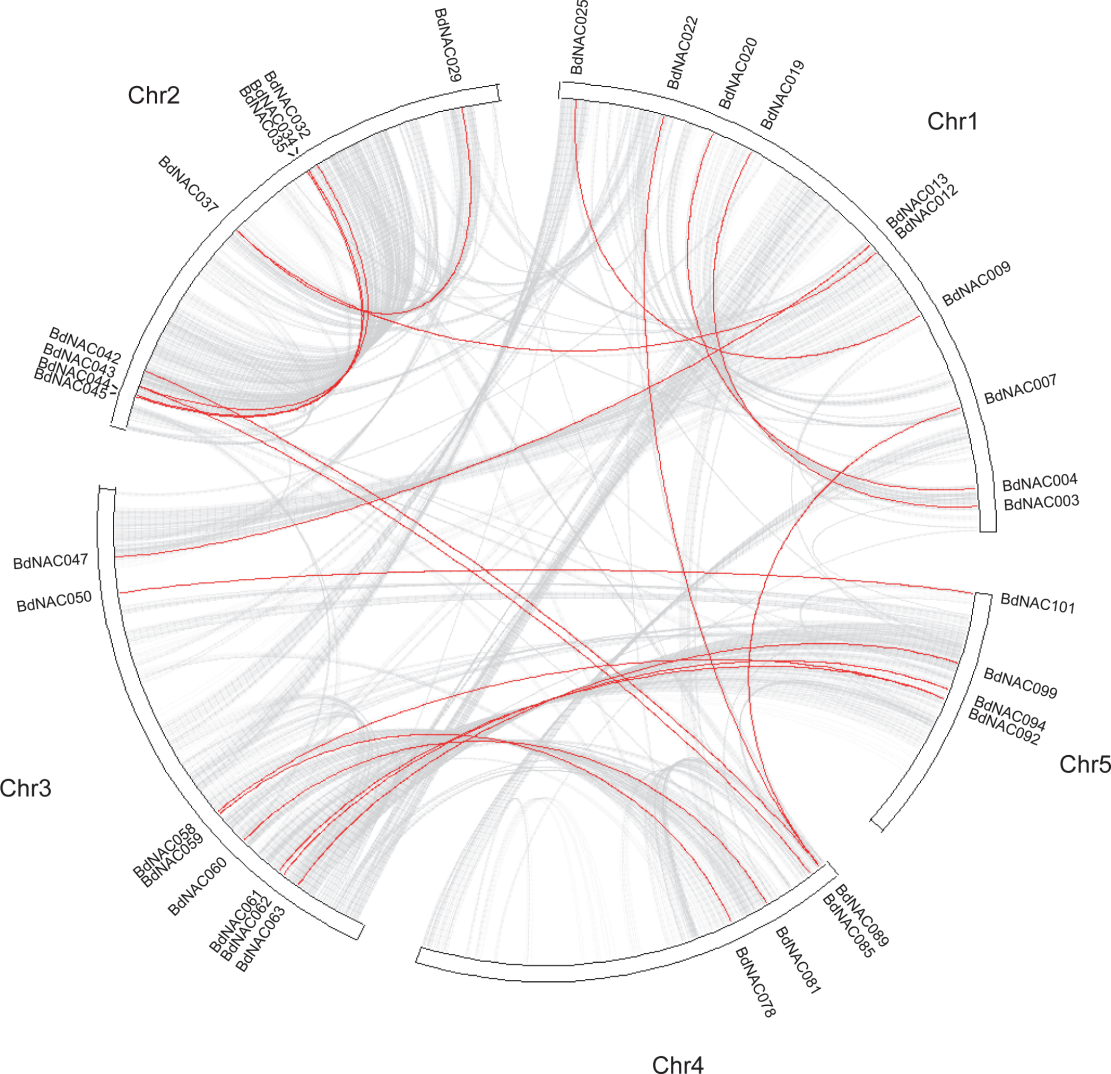
Circle plot showing segmentally duplicated *BdNAC* genes on 5 *B*. *distachyon* chromosomes. Grey lines indicated collinear blocks in whole *B*. *distachyon* genome, and red lines indicated duplicated *BdNAC* gene pairs.

Sequencing analysis of the *B*. *distachyon* genome revealed that chromosomal duplications cover 92.1% of the genome [27]. We further analyzed the tandem and segmental duplication events of *BdNAC* genes. According to the whole genome analysis in *B*. *distachyon*, 2945 (11.1%) and 4889 (18.2%) genes were identified as tandem and segmental duplications, respectively (S3 and S4 Tables). Among NAC family members in *B*. *distachyon*, 9 (8.9%) *BdNAC* genes were found to be tandem repeats. This includes 3 clusters of tandemly repeated *BdNACs* (*BdNAC001* and *BdNAC002*, *BdNAC070* and *BdNAC071*, and *BdNAC086* and *BdNAC087*). Besides the tandemly duplicated *BdNAC* genes, 35 (34.7%) segmentally duplicated *BdNAC* genes were also identified. As shown in Fig. 2, these *BdNAC* genes were located on duplicated segments on all 5 chromosomes. Maximum 10 *BdNACs* were located in duplicated segments on chromosomes 1.

### Structural and phylogenetic analysis of *BdNAC* genes

**Fig 3:**
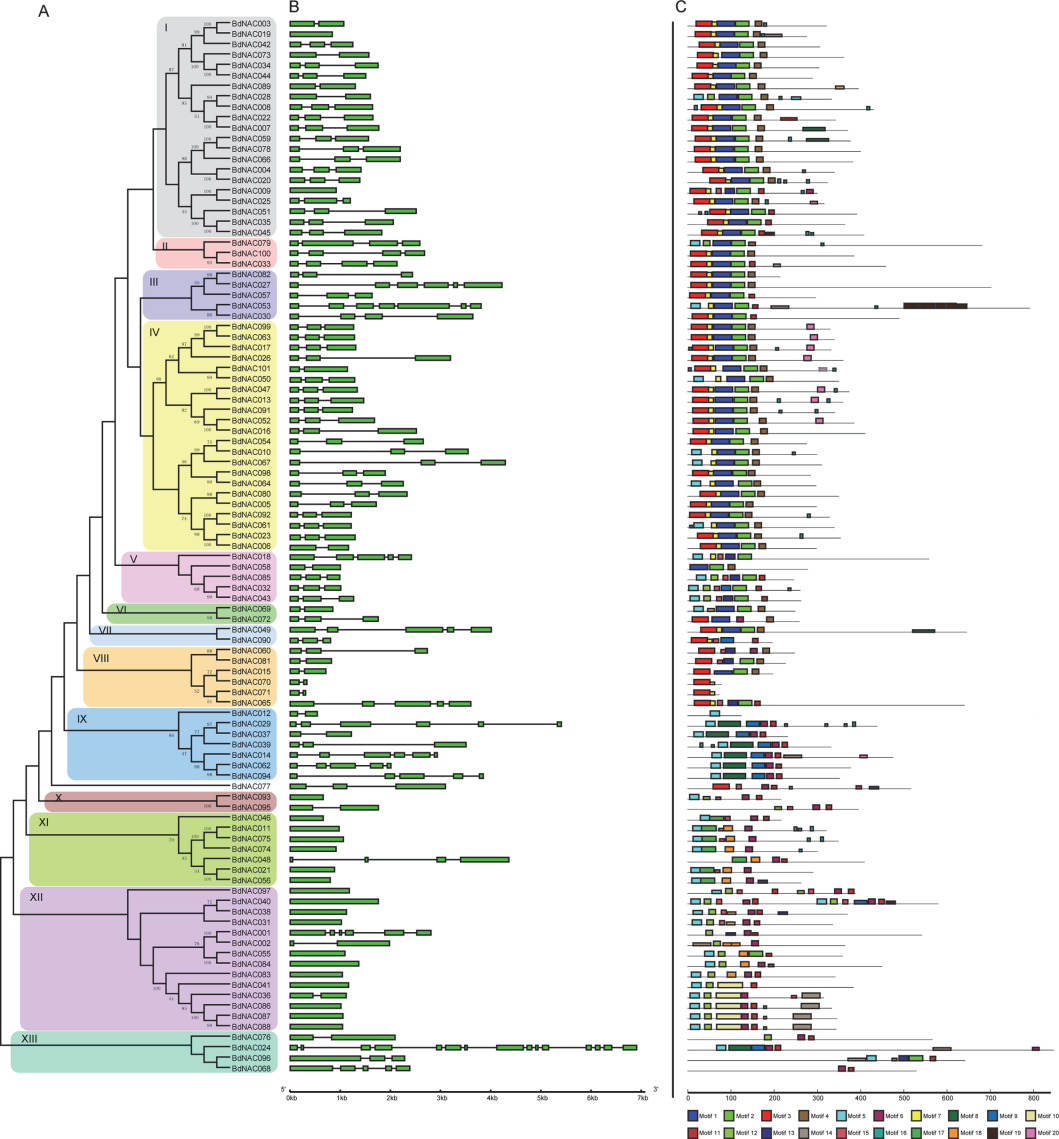
Phylogenetic relationships, gene structure and motif compositions of *BdNAC* genes. (A). The unrooted phylogenetic tree was created in MEGA5 software with the neighbor-joining method with 1,000 bootstrap iterations based on 101 full-length amino acids of BdNAC TFs. Thirteen major phylogenetic groups designated as I to XIII were marked with different color backgrounds. (B). Exon/intron structures of *BdNAC* genes. Exons and introns were represented by green boxes and black lines, respectively. Sizes of exons and introns could be estimated using the scale at bottom. (C). Schematic representation of the conserved motifs in the BdNAC TFs elucidated by MEME. Each motif was represented by a colored box numbered at the bottom. The black lines represented the non conserved sequences. The length of protein could be estimated using the scale at the bottom. The details of individual motif were shown in S5 Fig.

The identified *BdNAC* genes encode proteins ranging from 75 (BdNAC071) to 856 (BdNAC024) amino acids (aa) in length with an average of 368 aa. Multiple sequence alignment of BdNACs along with three representative Arabidopsis NAC proteins, such as NAM, ATAF1 and TIP showed that all the BdNAC proteins contained highly conserved N-terminal NAC domain (S2 Fig.). Most BdNACs have complete NAC domain that consists of five subdomains (A-E). However, BdNAC058 lacks conserved A and B subdomains, and three BdNACs (BdNAC012, BdNAC070 and BdNAC071) do not contain conserved C, D and E subdomains. These NAC proteins may be characterized as NAC-like proteins according to the description of these proteins in rice [8]. In addition to the NAC domain, other conserved protein domains and trans-membrane (TM) helices in BdNACs were also identified by Pfam and TMHHM, respectively (S5 Table and S3 Fig.). Based on the domain architecture, BdNACs are divided into five groups. Most of the BdNACs are typically NAC proteins that have N-terminal conserved NAC domain and a C-terminal variable region and were classified into structure group I. Three proteins (BdNAC012, BdNAC070 and BdNAC071) containing only the NAC domains were classified into structure group II. Structure group III BdNACs (BdNAC040 and BdNAC097) have two tandem repeated NAC domains. Multiple sequence alignment indicated that the NAC domain at the C-terminal end of BdNAC097 had great variations in subdomain A (S4 Fig.). Structure group IV with seven proteins (BdNAC018, BdNAC030, BdNAC049, BdNAC053, BdNAC065, BdNAC076, BdNAC079) were predicted to comprise a single TM region. Among these predicted trans-membrane BdNACs, only the TM region of BdNAC076 was located at N-terminal. Two BdNACs in structure group V contain other conserved protein domains in addition to the NAC domain. A BED zinc finger domain was identified in BdNAC068. A plant invertase/pectin methylesterase inhibitor domain presents in BdNAC076 indicating a putative involvement of this protein in regulation of cell wall extension. To investigate the evolutionary relationship among 101 BdNACs, an unrooted tree was constructed from alignments of full-length NAC protein sequences using MEGA 5.0 by the neighbor-joining (NJ) method. As shown in Fig. 3A, 101 BdNACs were divided into 13 distinct groups (designated group BdNAC-I to BdNAC-XIII). BdNAC077 was distinguished from other members and formed an individual clade. Group IV consists of the maximum number (22) of BdNACs, while group VI, VII and X each contains a minimum of two BdNACs. All tandem duplicated NAC genes were assigned to same groups with high bootstrap values, as reported in other plant species [8, 34].

The genomic sequence of the longest *BdNAC* gene (*BdNAC024*) was about 7.7 kb while the shortest one (*BdNAC071*) was only 309 bp. In order to gain further insights into the structural diversity of *BdNAC* genes, we compared the exon/intron organization in the coding sequences of *BdNAC* genes (Fig. 3B). Highly diverse distribution of intronic regions (from 0 to 14 in numbers) was found among *BdNAC* genes. In general, *BdNACs* clustered in the same group exhibit similar exon/intron structure in terms of intron number (Fig. 3B). All *BdNAC* genes in the group XI have no intron except *BdNAC048*, while most of *BdNAC* genes in group I and IV have two to three introns. In contrast, largest number of exon/intron structural variants was observed in the group IX and XIII. To further reveal the diversification of BdNACs, putative motifs were predicted by the program MEME. A total of 20 putative motifs were predicted in BdNACs, and the details of sequence logo of each motif were shown in S5 Fig. As expected, BdNACs in the same groups share similar motif composition (Fig. 3C), suggesting putatively functional similarity among members of the same group. Most of the conserved motifs were located in the N-terminal NAC domain, indicating most BdNACs contain conserved N-terminal NAC domain and diversified C-terminal region. In general, conserved N-terminal NAC domains of most BdNACs harbor five conserved motifs, which representing the five subdomains (A-E) of typical NAC domain. Noticeably, some specific motifs were presented in NACs from a specific group or member. For instance, motif 17 presents in six members of subgroup XI, and motif 19 only presents in BdNAC053 as 5 tandem repeats. Whether these motifs confer unique functional roles to BdNACs remained to be further investigated. Diversified domain architecture and organization of exon/intron structure and putative motifs suggested potential diverse functions of NAC family in *B*. *distachyon*.

### Phylogenetic analysis of NAC genes in *B*. *distachyon*, Arabidopsis and rice

To further analyze evolutionary relationships in the NAC genes family, BdNACs and NACs from the eudicot (Arabidopsis) and monocot (rice) model systems were subjected to comprehensive phylogenic analysis. Nine NACs from rice and Arabidopsis were removed due to great variation of their full-length protein sequences (see method). Thus a total of 332 NACs, comprising 99 from Arabidopsis, 132 from rice, and 101 from *B*. *distachyon*, were used for construction of an unrooted phylogenetic tree. As illustrated in S6 Fig., the phylogenetic analysis classified the BdNACs into several groups together with their Arabidopsis and rice orthologs. The groups were designated as alphabetical families (NAC-a to NAC-p) based on tree topologies. Group NAC-f constituted the largest clade, containing 50 members and accounting for 15% of the total NACs, while group NAC-b formed the second largest clade containing 47 members and accounting for 14% of the total NACs. Noticeably, among the groups identified by phylogenetic analysis, two groups (NAC-f and NAC-m) only contain NACs from rice and *B*. *distachyon*, and group NAC-g only contains Arabidopsis NACs. This result suggests diversification and expansion of these subgroups after the monocot-eudicot split.

As reviewed in the introduction, NAC proteins have been demonstrated to be involved in diverse aspects of plant growth, development, and stress responses. However, the functions of most members in this family remain unknown. Out of the 101 BdNACs identified in this study, only BdNAC101/BdSWN5 has been characterized to function in regulation of secondary wall synthesis and cell death [29]. Therefore, prediction of the function of BdNACs through homologous analysis is important for the future study of NACs in *B*. *distachyon*. For example, group NAC-a encompassed the NAC proteins involved in secondary wall synthesis. These NACs include NSTs (NST1, NST2, NST3/SND1) and VNDs (VND1-7) from Arabidopsis and OsSWNs (OsSWN1-7) from rice [19]. Moreover, *B*. *distachyon* NAC protein BdNAC101/BdSWN5 in group NAC-a has a similar function for regulation of secondary wall synthesis [29], suggesting that phylogeny-based functional prediction is helpful for NACs functional characterization in *B*. *distachyon* as that in rice and soybean [8, 9].

**Fig 4:**
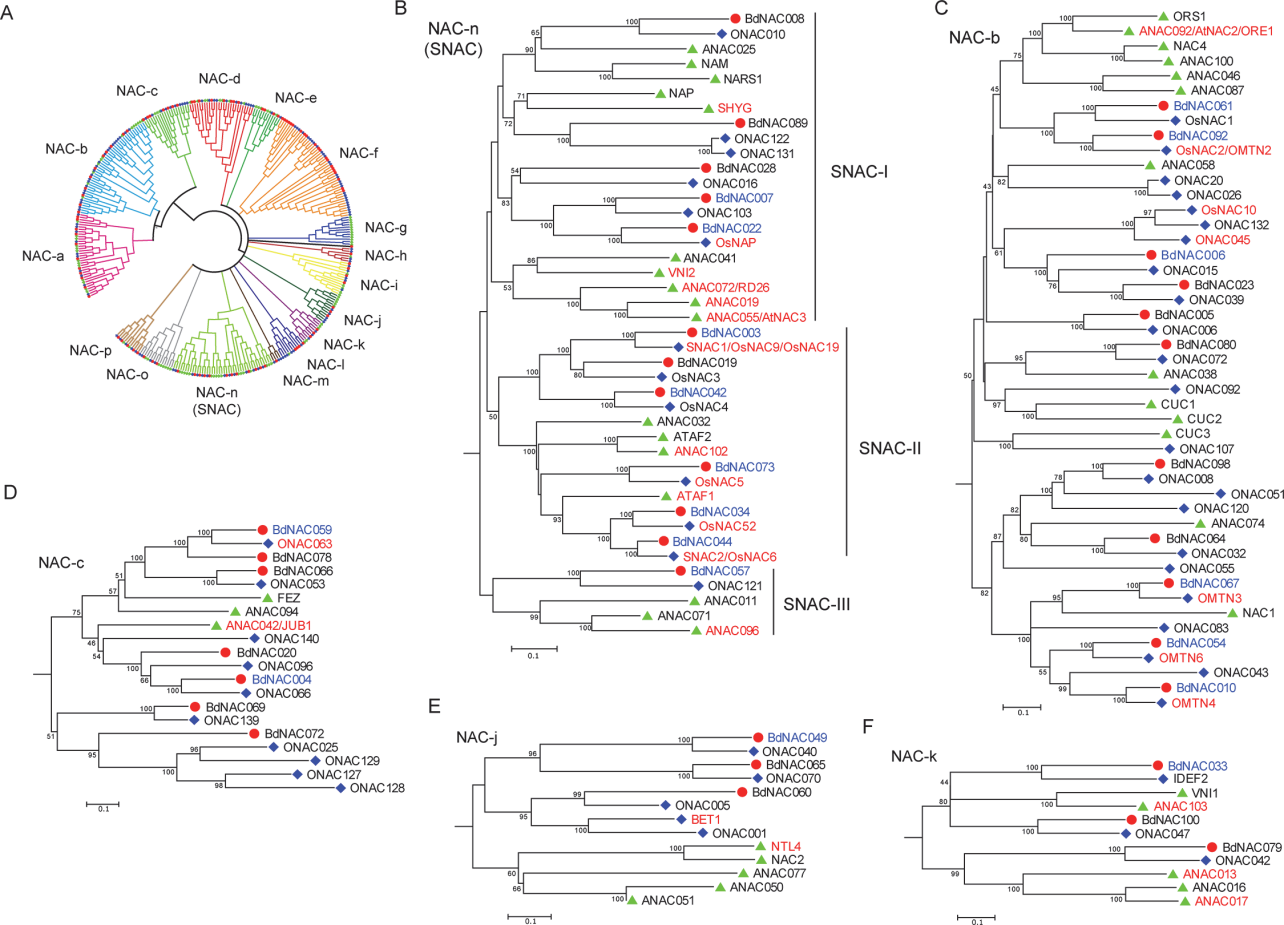
Phylogenetic analysis-based prediction of abiotic stress-related *BdNAC* genes. (A). Phylogenetic relationship of NAC proteins from *B*. *distachyon*, Arabidopsis and rice. The unroofed phylogenetic tree was constructed using the full-length of 332 NAC proteins from *B*. *distachyon*, Arabidopsis and rice by MEGA5. Only the tree topology was presented. The detailed unroofed phylogenetic tree was shown in S6 Fig. (B-F). Clades enriched with stress-related NACs including NAC-n (B), NAC-b (C), NAC-c (D), NAC-j (E) and NAC-k (F). Group NAC-n contains most stress-related NACs (also named it as SNAC), and can be further divided into three subgroups (SNAC-I, SNAC-II and SNAC-III). NAC proteins from *B*. *distachyon*, Arabidopsis and rice were denoted by red circle, green triangle and blue diamond, respectively. Known stress-responsive *NAC* genes from Arabidopsis and rice were indicated in red. Putative stress-related *BdNAC* genes based on phylogenetic analysis were indicated in blue.

The main purpose of this study was to identify the putative *BdNAC* genes that function in abiotic stress responses. Previous reports of phylogenetic analysis of NAC family found that many stress-responsive NACs are in the SNAC (stress-responsive NAC) group [8]. Our phylogenetic analysis revealed that group NAC-n contains most stress-related NACs, and named it as SNAC (Fig. 4). Out of 42 NACs in SNAC group, 13 (31%) NACs have been demonstrated their functions in abiotic stress responses (indicated in red in Fig. 4). SNAC group can be further divided into three subgroups: SNAC-I, SNAC-II and SNAC-III (Fig. 4B). Senescence is the common consequence of various abiotic stresses. Most members of SNAC-I subgroup have a dual function in abiotic stress responses and senescence regulation, such as ANAC019, ANAC055/AtNAC3, ANAC072/RD26 and VNI2 from Arabidopsis [14, 21, 35, 36], and OsNAP from rice [37–39]. SNAC-II subgroup contains several well known stress-related NAC genes from Arabidopsis and rice. For example, SNAC1/OsNAC9/OsNAC19 and OsNAC5 enhanced drought and salt resistances [22, 25], while SNAC2/OsNAC6 increased salt and cold resistances in transgenic rice [23, 24]. ATAF1 from Arabidopsis also enhanced plant resistance to drought [40]. In addition to their functions in abiotic stress responses, most members of SNAC-II subgroup, such as SNAC1/OsNAC9/OsNAC19, SNAC2/OsNAC6 and ATAF1, were also involved in biotic stress responses [23, 40, 41]. ATAF2 and OsNAC4 in SNAC-II subgroup play a role in biotic stress response [42, 43], but their functions in abiotic stresses remain unknown. ANAC096 in SNAC-III subgroup functioned in dehydration and osmotic stress responses through direct interaction with ABF2 and ABF4 [44]. In addition to SNAC group, abiotic stress-related NACs were also mainly distributed in group NAC-b, NAC-c, NAC-j and NAC-k (Fig. 4C-F). NAC-b contains NACs functioning in development (such as CUC1-3). Recently, some members were found to be involved in abiotic stress responses. ORE1/ANAC092/AtNAC2 plays an important role in salt-promoted senescence [45]. Jeong et al. [26] reported that overexpression of *OsNAC10* increased grain yield significantly under field drought condition. Fang et al. [46] reported that four miR164-targeted NAC genes (*OMTN2*, *3*, *4* and *6*) negatively regulated drought resistance in rice. JUB1/ANAC042 in group NAC-c delayed senescence and enhanced resistance to various abiotic stresses by modulating cellular H_2_O_2_ level and DREB2A regulated network [18]. Membrane-bound NAC transcription factor NTL4 in group NAC-j promoted production of reactive oxygen species during drought-induced leaf senescence in Arabidopsis [20]. Two membrane-bound NAC transcription factors in group NAC-k, ANAC013 and ANAC017, were direct positive regulators of *AOX1a* and functioned in mitochondrial retrograde regulation of the oxidative stress response in Arabidopsis [47, 48]. In summary, a total of 36 *BdNAC* genes were clustered into five groups which were enriched with abiotic stress-related NACs, and 18 of them with highly homologous to known stress-related NAC TFs from Arabidopsis and rice were identified as putative stress-related *BdNAC* genes (indicated in blue in Fig. 4).

### *In silico* stress-related *cis*-element analysis of *BdNAC* genes

To identify putative stress-responsive *cis*-elements in the promoter regions of the *BdNAC* genes, 1kb upstream promoter sequences of the *BdNAC* genes were subjected to search against the PlantCARE database (http://bioinformatics.psb.ugent.be/webtools/plantcare/html/) [49, 50]. Totally, 5 types of stress-related *cis*-elements were detected, including MYB binding site involved in drought-inducibility (MBS), dehydration-responsive element (DRE), low temperature-responsive element (LTR), heat shock element (HSE), and defense and stress-responsive element (TC-rich repeats). In addition, some elements possibly participated in response to hormones, such as ABA, Eth, methyl jasmonate (MeJA), and SA, were also identified. All of the 101 *BdNAC* genes contain at least one *cis*-element related to stress or hormone responses, and 44 *BdNAC* genes had more than 5 *cis*-elements, suggesting these *BdNAC* genes might be involved in the stress- or hormones-response processes (S6 Table). For example, we found 12, 11 and 10 *cis*-elements in the promoters of *BdNAC073*, *BdNAC014* and *BdNAC044*, respectively. In addition, 135 ABA-responsive elements (ABREs), 107 CGTCA-motifs (MeJA-responsiveness), 99 MBSs, 64 TCA-elements (SA-responsiveness) and 51 TC-rich repeats were detected in the promoters of *BdNAC* genes, suggesting that *BdNAC* genes have important roles in the responses to ABA, MeJA, drought, SA and defense responses (S6 Table). Among 101 *BdNAC* genes, 60, 40, 34 and 32 *BdNAC* genes had MBSs, TC-rich repeats, LTRs and HSEs, respectively (S6 Table), while only 3 *BdNAC* genes contain DREs in their promoter regions. BdNAC002, BdNAC048, BdNAC055, BdNAC068, BdNAC070 and BdNAC083 might be possibly involved in response to multiple stresses because of the enrichment of various stress-related *cis*-elements, such as MBSs, LTRs and HSEs. Approximately 62%, 58% and 49% of all *BdNAC* genes had MeJA-responsive elements, ABREs and SA-responsive elements in their promoter regions, respectively (S6 Table). Furthermore, 14 *BdNAC* genes contain 4 to 10 ABA-, MeJA- or SA-responsive elements in their promoter regions. Especially, *BdNAC034*, *BdNAC014*, *BdNAC086*, *BdNAC039* and *BdNAC085* contain over 5 copies of ABREs in their promoters, suggesting that these *BdNAC* genes might be possibly involved in stress responses through ABA signal pathway.

### Expression profiles of *BdNAC* genes in response to abiotic stresses

**Fig 5:**
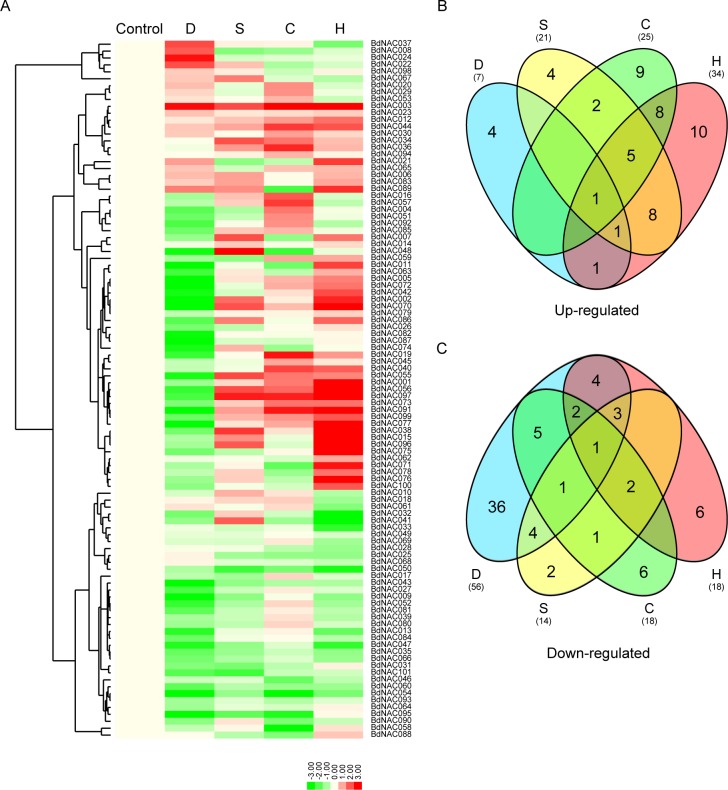
Expression profile of *BdNAC* genes in response to various abiotic stresses. (A). Hierarchical clustering of expression profile of *BdNAC* genes in response to drought (D), salinity (S), cold (C) and heat (H) stresses. Three-week-old seedlings were subjected to drought stress (growth without water supply), salt (200 mM NaCl), cold (4°C) and heat (42°C) stresses. Relative expression levels of the *BdNAC* genes were analyzed by quantitative real-time RT-PCR (qPCR), and log2-transformed fold-change values were used for creating the heatmap (original data were shown in S7 Fig.). Venn diagram illustrated the distribution of the up-regulated (B) or down-regulated (C) *BdNAC* genes response to different abiotic stresses. The common subset of genes regulated by two or more stresses was marked by the overlapping circle.

A number of NAC proteins have been demonstrated to play important roles in abiotic stress responses in plants [5]. To obtain an overview of the stress responses of the BdNAC family, the transcript levels of all 101 *BdNAC* genes were investigated in seedlings under multiple stress conditions including drought (growth without water supply), salinity (200 mM NaCl), cold (4°C) and heat (42°C) treatments. Detailed expression profiles of the *BdNAC* genes under different stress conditions were presented in S7 Fig. Heat map representation for transcript expression fold change in response to abiotic stresses was shown in Fig. 5A. Based on the cluster analysis, the expression of 6 *BdNAC* genes was induced by drought but repressed by heat stress, and 59 *BdNAC* genes were induced by multiple stresses except drought stress, while other 36 *BdNAC* genes were mainly repressed by all four abiotic stresses. More than 50% of the *BdNAC* genes were down-regulated under drought stress condition. Whereas, 34 (33%) *BdNAC* genes were up-regulated under heat stress condition. Among the 101 *BdNAC* genes, 53 and 73 genes were up-regulated and down-regulated under at least one stress condition, respectively (Fig. 5B and C). Additionally, 26 and 23 *BdNAC* genes were up-regulated and down-regulated under more than one stress condition, respectively. For examples, *BdNAC034* and *BdNAC036* were up-regulated by both salinity and cold treatments, while *BdNAC013*, *BdNAC032*, *BdNAC052* and *BdNAC066* were down-regulated by both drought and heat treatments. Some of the *BdNAC* genes exhibited induction under specific stress condition, for example, *BdNAC008*, *BdNAC022*, *BdNAC024* and *BdNAC037* were induced specifically under drought stress condition, while *BdNAC006*, *BdNAC041*, *BdNAC048* and *BdNAC067* were up-regulated only by high salinity treatment. These results indicated that BdNACs might regulate complex networks to cope with multiple adverse environmental conditions.

### Expression profiles of *BdNAC* genes in responsive to hormone treatments

**Fig 6:**
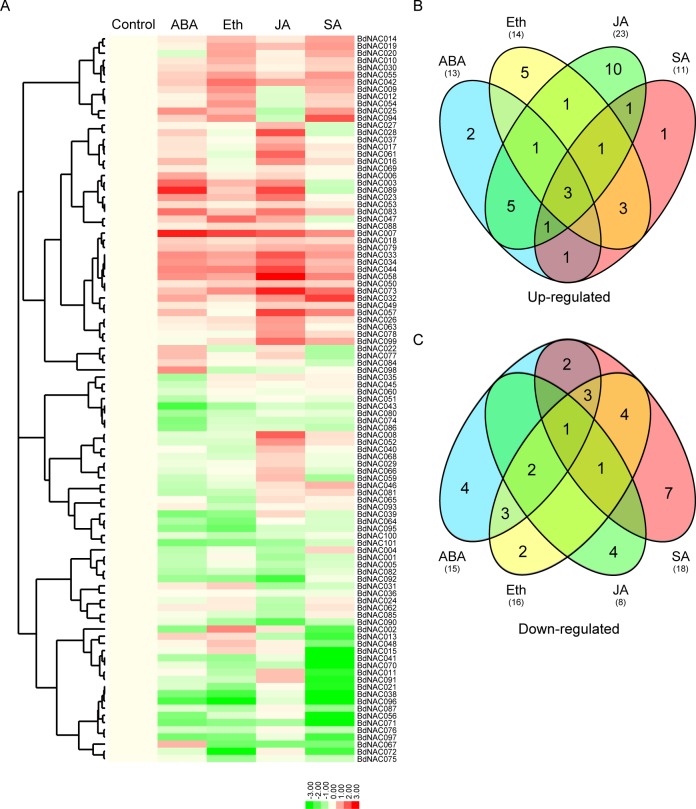
Expression profile of *BdNAC* genes in response to various phytohormones. (A). Hierarchical clustering of expression profile of *BdNAC* genes in response to abscisic acid (ABA), ethephone (Eth), jasmonic acid (JA) and salicylic acid (SA) treatments. Three-week-old seedlings were treated with 100 μM ABA, 300 μM ethephon, 100 μM JA and 100 μM SA, respectively. Relative expression levels of the *BdNAC* genes were analyzed by quantitative real-time RT-PCR (qPCR), and log2-transformed fold-change values were used for creating the heatmap (original data were shown in S8 Fig.). Venn diagram illustrated the distribution of the up-regulated (B) or down-regulated (C) *BdNAC* genes response to different phytohormone treatments. The common subset of genes regulated by two or more phytohormones was marked by the overlapping circle.

To identify hormone-responsive *BdNAC* genes, *B*. *distachyon* Bd21 seedlings were treated with ABA, Eth, JA and SA, respectively, and the changes in transcript abundance of all the 101 *BdNAC* genes were analyzed (S8 Fig.). Hierarchical clustering showed overlapping and specific gene expression patterns in response to phytohormones (Fig. 6A). A total of 13, 14, 23 and 11 *BdNAC* genes were up-regulated by ABA, Eth, JA and SA treatments, respectively. Whereas, 15, 16, 8 and 18 *BdNAC* genes were down-regulated by ABA, Eth, JA and SA treatments, respectively (Fig. 6B and C). More than 50% of the *BdNAC* genes were repressed by most of exogenous phytohormone treatments. Of the 101 *BdNAC* genes, 35 and 33 genes were up- and down-regulated by at least one phytohormone treatment, respectively (Fig. 6B and C). Additionally, 17 and 16 *BdNAC* genes were up- and down-regulated by more than one phytohormone treatment, respectively. Some *BdNAC* genes were induced (*BdNAC007*, *BdNAC033* and *BdNAC058*) or repressed (*BdNAC071*) by all four phytohormone treatments. These expression profiles suggest a divergence in the functions of *BdNAC* genes in different hormone signal pathways.

## Discussion

The NAC proteins are characterized by a conserved DNA-binding domain and constitute one of the largest families of transcription factors in plants. As NAC TFs are plant-specific proteins, we also searched NAC proteins in six major types of model organisms whose genomes have been already sequenced, including red alga (*Cyanidioschyzon merolae*), the chlorophytes (*Ostreococcus lucimarinus*, *Ostreococcus tauri*, *Coccomyxa subellipsoidea*, *Chlamydomonas reinhardtii*, and *Volvox carteri*), the moss (*Physcomitrella patens*), the lycophyte (*Selaginella moellendorffii*), the monocots (*Oryza sativa*, *Brachypodium distachyon*, *Sorghum bicolor*, and *Zea mays*), and the eudicots (*Solanum lycopersicum*, *Vitis vinifera*, *Arabidopsis thaliana*, *Glycine max*, and *Populus trichocarpa*). The result showed that the plant-specific NAC genes do not exist in unicellular or multicellular algae, and only found in land plants (S9 Fig.). Small number of NAC genes has been found in moss and lycophyte, while angiosperms possess a large number of NAC genes. The results indicated that the NAC TFs may arise after plants transitioned from water to land, and are particularly important for plant tissue organization and adverse environmental adaptation. More than 100 NAC genes were identified in most angiosperms, while *B*. *distachyon* and tomato had the fewest except grape. The presence of such large number of NACs in plants indicated great functional diversity and amplification of this gene family during evolution.

In this study, 101 *BdNAC* genes were identified in the *B*. *distachyon* genome. The 101 *BdNAC* genes are unevenly distributed on all the 5 chromosomes of *B*. *distachyon*, and nine clusters each with 2 *BdNACs* were identified (Fig. 1). Uneven and cluster distribution of NAC family genes was also found in rice, poplar, foxtail millet, potato and soybean [9, 10, 34, 51, 52]. NAC membrane-bound TFs (MTFs) have been implicated in plant response to various abiotic stress conditions [20, 53]. Genome-wide analysis identified 18 NAC MTFs (NTLs) in Arabidopsis and 5 NAC MTFs (OsNTLs) in rice. Seven NAC proteins containing single TM were also identified in *B*. *distachyon*. All the Arabidopsis and rice NAC MTFs contain single TM at their C-terminal. However, one of the BdNAC MTFs, BdNAC076, had TM region at N-terminal (S3 Fig.). Segmental duplication, tandem duplication and transposition events were the main reasons for gene family expansion. 9 (8.9%) and 35 (34.7%) *BdNAC* genes were found to be tandem and segmental duplications, respectively. While, at whole genome level, 2945 (11.1%) and 4889 (18.2%) genes were identified as tandem and segmental duplications, respectively. These observations are consistent with a preferential retention of transcription factors after whole genome duplications [54].

Many NAC proteins are implicated in diverse plant developmental and physiological processes, including root development [16], leaf senescence [18], secondary walls formation [19], as well as various abiotic and biotic stress responses [22, 40, 44]. We are particularly interested in identification of NAC genes involving in response to abiotic stresses in *B*. *distachyon*. Phylogenetic analysis of NAC gene family in rice found that most of the stress-responsive NACs belonged to one group (namely stress-responsive NAC group, SNAC) [8]. Our phylogenetic analysis of NACs from *B*. *distachyon*, Arabidopsis and rice revealed that group NAC-n contains most stress-related NACs (named it as SNAC), and the SNAC group can be further divided into three subgroups (SNAC-I, SNAC-II and SNAC-III). On the basis of sequence alignments and phylogenetic analyses, 18 *BdNACs* with highly homologous to known stress-related NAC proteins from Arabidopsis and rice were identified as putative stress-related *BdNAC* genes (Fig. 4). Moreover, *cis*-elements and expression profiles analysis of *BdNAC* genes provided further evidence for their roles in stress tolerance. A total of 5 stress-related *cis*-elements were detected in the promoter regions of the *BdNAC* genes by search against the PlantCARE database, and 92 out of the 101 *BdNAC* genes had at least one of the stress-responsive *cis*-elements. Our qPCR results showed that the expression of 90% of all *BdNAC* genes were response to at least one of the stress conditions, suggesting that these *cis*-elements may play important roles in regulating gene expression in response to abiotic stresses. It is remarkable that the expressions of *BdNAC003*, ortholog of rice *SNAC1* genes, was highly induced by drought, high salinity, cold and heat stresses among the 18 putative stress-related *BdNAC* genes (Fig. 5 and S10 Fig.). And the promoter of *BdNAC003* contains stress-responsive *cis*-elements such as DRE and HSE. Whereas, rice *SNAC2* ortholog, *BdNAC044*, which has MBS and LTR, was induced by salinity, cold and heat stresses. Many *BdNAC* genes were down-regulated under abiotic stress conditions, indicating that these proteins might act as negative regulators in stress responses. Some NAC proteins have been reported for their negative regulatory roles in response to abiotic stresses. For example, four miR164-targeted NAC genes (*OMTN2*, *3*, *4* and *6*) were characterized as negative regulators of drought resistance in rice [46]. In this report, we found that *BdNAC054* and *BdNAC092*, ortholog of *OMTN6* and *OMTN2*, respectively, were substantially down-regulated by drought stress (Fig. 5 and S10 Fig.). These results indicated that function of some NAC proteins might be conserved among species. About half of the 18 candidate stress-related *BdNAC* genes, which contain at least one of the phytohormone-responsive *cis*-elements, were induced by ABA and other phytohormone treatments (Fig. 6 and S10 Fig.), indicating that *BdNACs* may function in abiotic stress response medicated by phytohormones such as ABA. The functions of these stress-responsive *BdNAC* genes in abiotic stress resistance will be further characterized in subsequent work.

Taken together, NAC family was comprehensive characterized in *B*. *distachyon* in this study and several candidate stress-responsive *BdNAC* genes were identified based on the phylogenetic and expression profile analyses. Our results presented here would be helpful in laying the foundation for understanding of the complex mechanisms of abiotic stress signaling controlled by NAC proteins in *B*. *distachyon*.

## Supporting Information

S1 Fig Genomic view showing location of *BdSWN1* in chromosome 1 of *B*. *distachyo*n. (PDF)

S2 Fig Identification of conserved NAC subdomains in BdNAC TFs by sequence analysis. (PDF)

S3 Fig Domain architecture of BdNAC TFs. (PDF)

S4 Fig Comparison of two tandem repeated NAC domain from BdNAC040 and BdNAC097. (PDF)

S5 Fig Sequence logos for conserved motifs identified in BdNAC TFs by MEME analysis. (PDF)

S6 Fig Phylogenetic tree of NAC TFs from *B*. *distachyon*, Arabidopsis and rice. (PDF)

S7 Fig Expression profile of *BdNAC* genes in response to various abiotic stresses. (PDF)

S8 Fig Expression profile of *BdNAC* genes in response to various phytohormones. (PDF)

S9 Fig Distribution of the NAC TFs in Plantae. (PDF)

S10 Fig Confirm the expression profiles of selected *BdNAC* genes under various abiotic stresses and phytohormone treatments by quantitative real-time RT-PCR with three biological repeats. (PDF)

S1 Table List of primers used for quantitative real-time RT-PCR analysis. (XLS)

S2 Table Spreadsheet of 101 NAC genes in *Brachypodium distachyon*. (XLS)

S3 Table List of segmentally duplicated gene pairs in *Brachypodium distachyon* genome along with their e-value identified from MCScanX software. (XLS)

S4 Table List of tandem and segmental duplicated gene in *Brachypodium distachyon* genome identified from MCScanX software. (XLS)

S5 Table Structure of NAC TFs in *Brachypodium distachyon*. (XLS)

S6 Table Numbers of known stress-related elements in the promoter regions of *BdNAC* genes. (XLS)
