# Supplementary material for: Systematic Analysis and Identification of Stress-Responsive Genes of the NAC Gene Family in Brachypodium distachyon
Source: PLoS One. 2015 Mar 27;10(3):e0122027. doi: 10.1371/journal.pone.0122027 (PMC4376915; doi:10.1371/journal.pone.0122027)
Supplement: S1 Fig — (PDF) [file pone.0122027.s001.pdf]

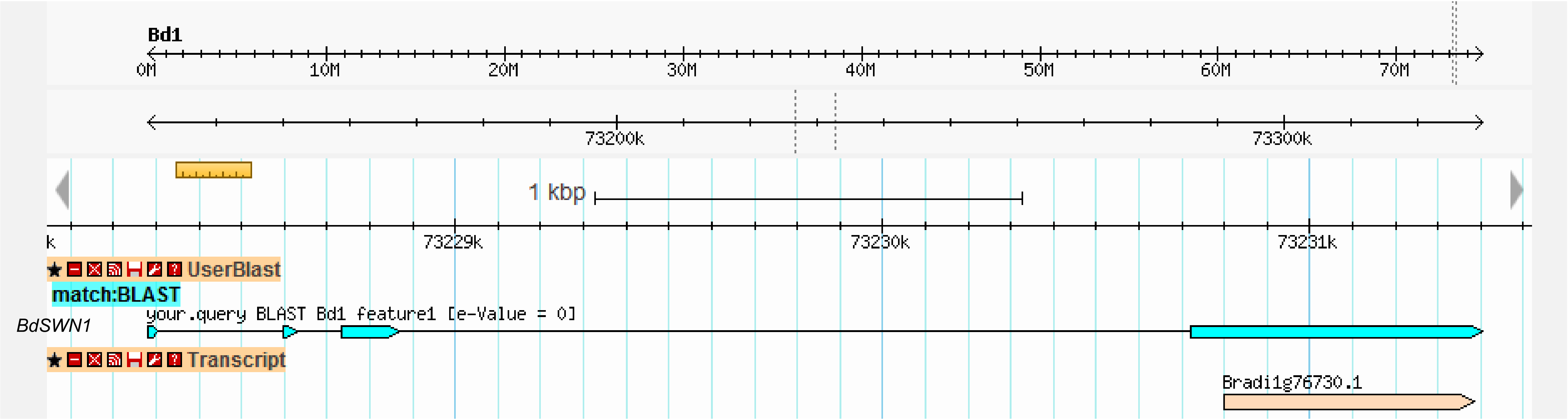

S1 Fig. Genomic view showing location of *BdSWN1* in chromosome 1 of *B. distachyon*. Nucleotide sequence of *BdSWN1* (Accession number: JQ693422) was blast against *B. distachyon* annotation database at Phytozome v9.1 indicated incorrect annotation of locus Bradi1g76730.
