# Supplementary figures and images for: Systematic Analysis and Identification of Stress-Responsive Genes of the NAC Gene Family in Brachypodium distachyon
Source: PLoS One. 2015 Mar 27;10(3):e0122027. doi: 10.1371/journal.pone.0122027 (PMC4376915; doi:10.1371/journal.pone.0122027)

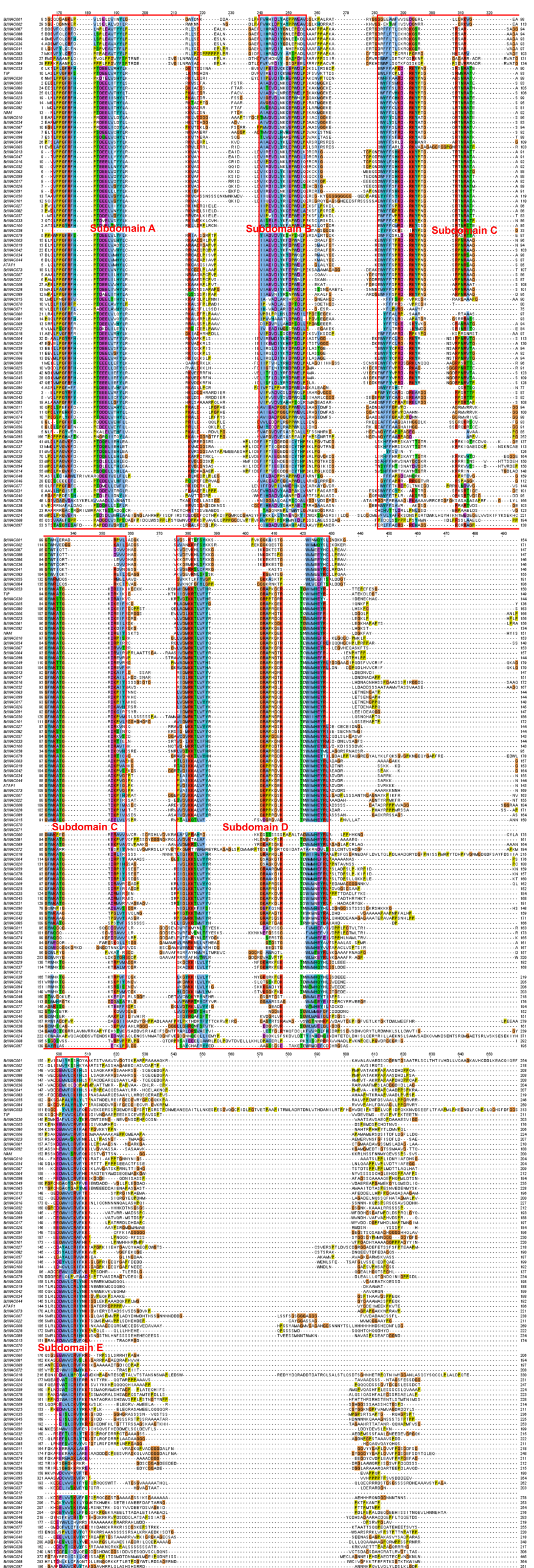

Supplement: S2 Fig — (PDF) [file pone.0122027.s002.pdf]
