# Supplementary material for: Systematic Analysis and Identification of Stress-Responsive Genes of the NAC Gene Family in Brachypodium distachyon
Source: PLoS One. 2015 Mar 27;10(3):e0122027. doi: 10.1371/journal.pone.0122027 (PMC4376915; doi:10.1371/journal.pone.0122027)
Supplement: S3 Fig — (PDF) [file pone.0122027.s003.pdf]

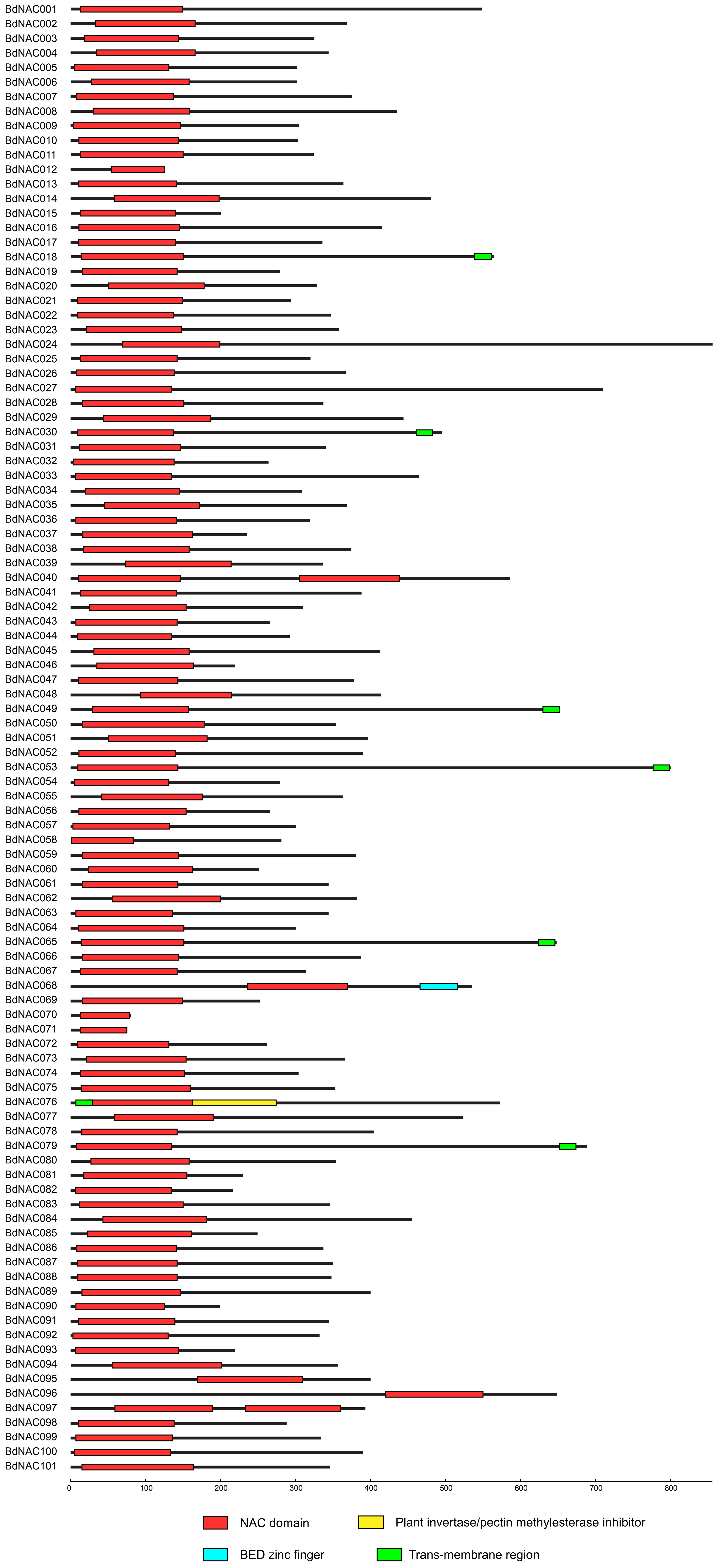

S3 Fig. Domain architecture of BdNAC TFs. Conserved domain in the *B. distachyon* NAC TFs were elucidated by Pfam. Each domain is represented by a colored box. The black lines represent the non conserved sequences. The length of protein can be estimated using the scale at the bottom.
