# Supplementary material for: Systematic Analysis and Identification of Stress-Responsive Genes of the NAC Gene Family in Brachypodium distachyon
Source: PLoS One. 2015 Mar 27;10(3):e0122027. doi: 10.1371/journal.pone.0122027 (PMC4376915; doi:10.1371/journal.pone.0122027)
Supplement: S5 Fig — (PDF) [file pone.0122027.s005.pdf]

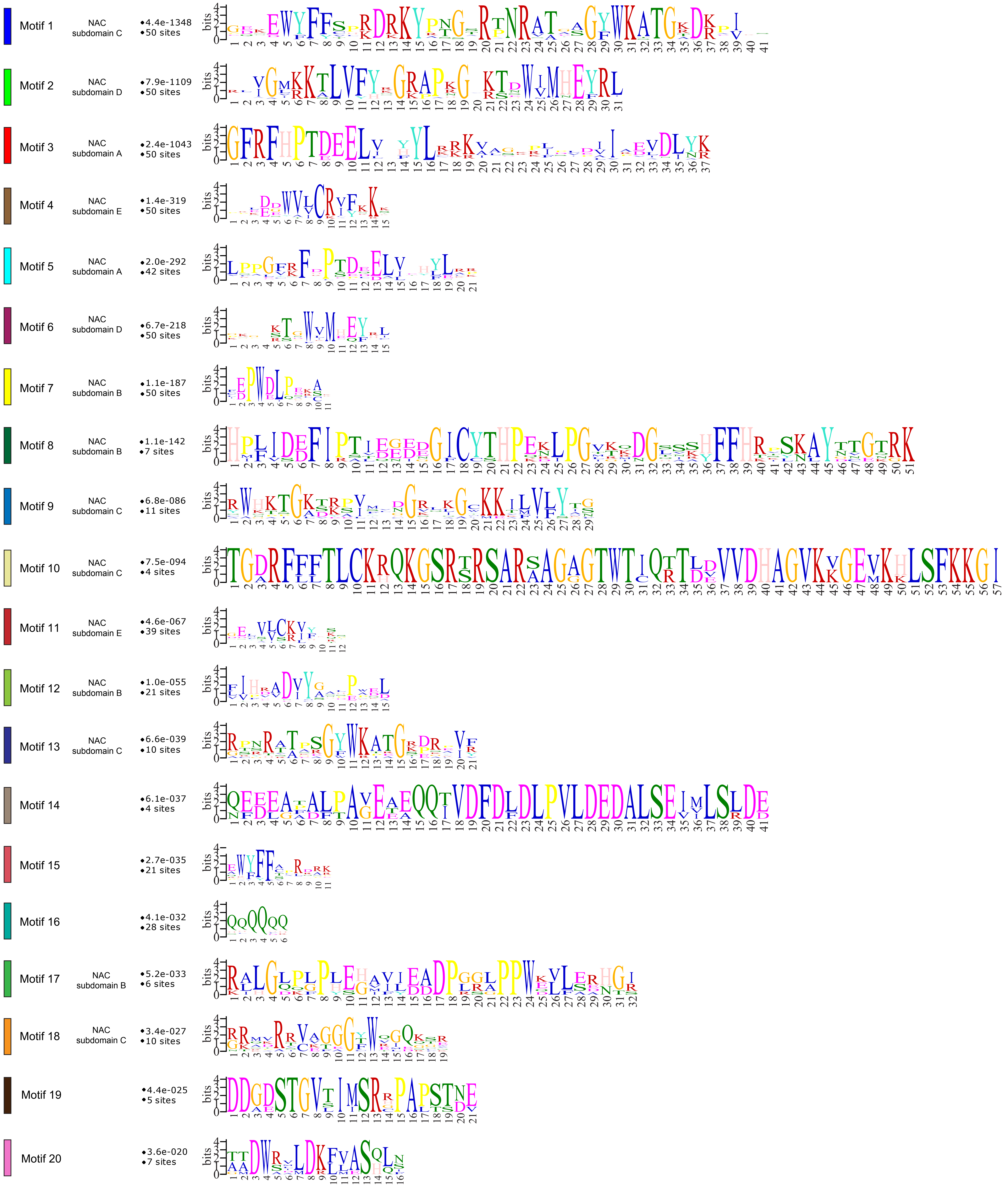

S5 Fig. Sequence logos for conserved motifs identified in BdNAC TFs by MEME analysis. Motif 3 and 5 represents the NAC subdomain A, motif 7, 8, 12 and 17 represents the NAC subdomain B, motif 1, 9, 10 and 13 represents the NAC subdomain C, motif 2 and 6 represents the NAC subdomain D, and motif 4 and 11 represents the NAC subdomain E.
