# Supplementary material for: Systematic Analysis and Identification of Stress-Responsive Genes of the NAC Gene Family in Brachypodium distachyon
Source: PLoS One. 2015 Mar 27;10(3):e0122027. doi: 10.1371/journal.pone.0122027 (PMC4376915; doi:10.1371/journal.pone.0122027)
Supplement: S6 Fig — (PDF) [file pone.0122027.s006.pdf]

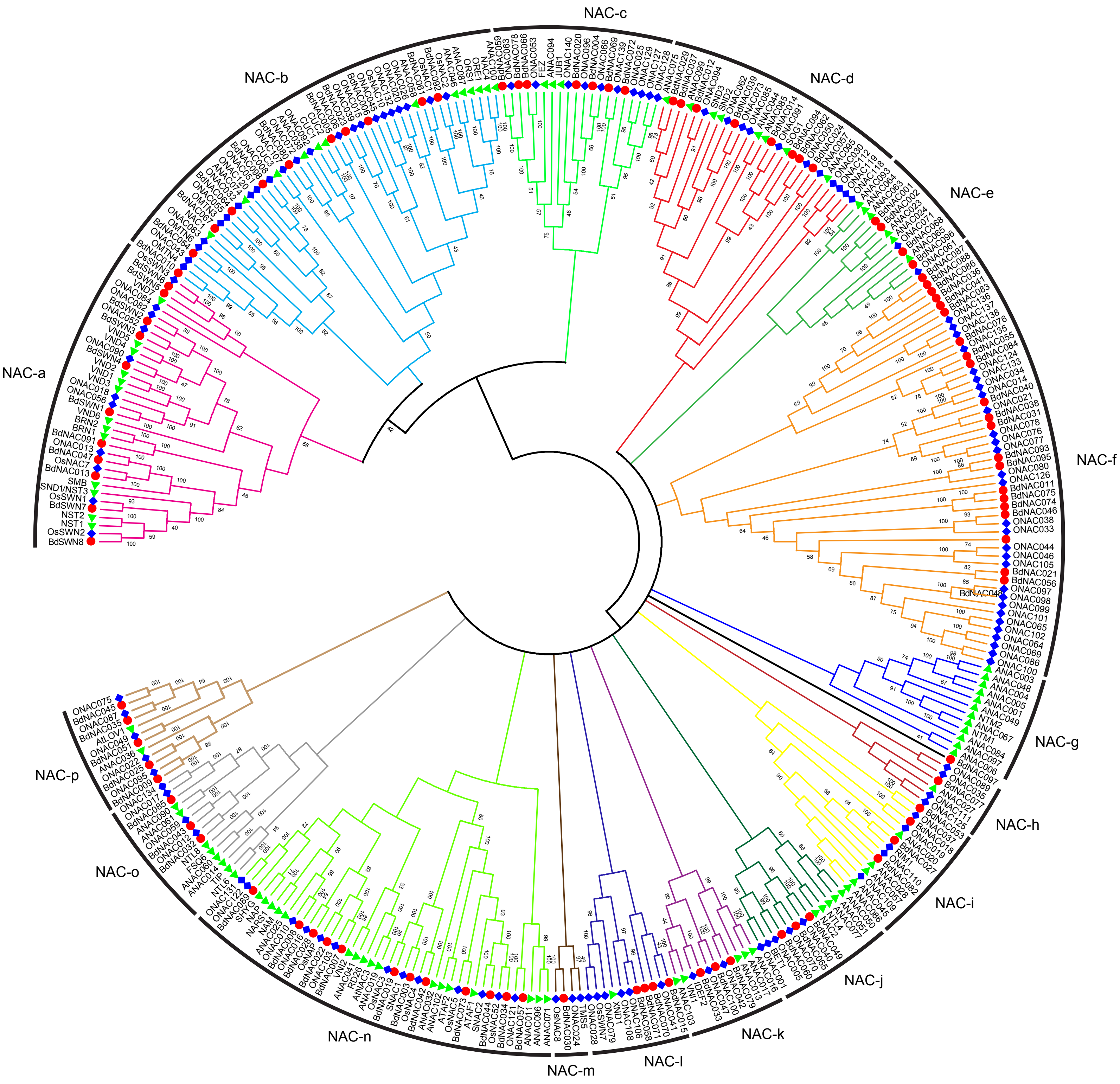

S6 Fig. Phylogenetic tree of NAC TFs from *B. distachyon*, Arabidopsis and rice. The deduced full-length amino acid sequences of 101 NAC TFs in *B. distachyon*, 99 in Arabidopsis and 132 in rice were used to construct an unrooted phylogenetic tree using MEGA5 by the neighbor-joining method with 1000 bootstrap iterations. Each NAC group was indicated in a specific color. Members of *B. distachyon*, Arabidopsis and rice were denoted by red circle, green triangle and blue diamond, respectively.
