# Supplementary material for: Systematic Analysis and Identification of Stress-Responsive Genes of the NAC Gene Family in Brachypodium distachyon
Source: PLoS One. 2015 Mar 27;10(3):e0122027. doi: 10.1371/journal.pone.0122027 (PMC4376915; doi:10.1371/journal.pone.0122027)
Supplement: S7 Fig — (PDF) [file pone.0122027.s007.pdf]

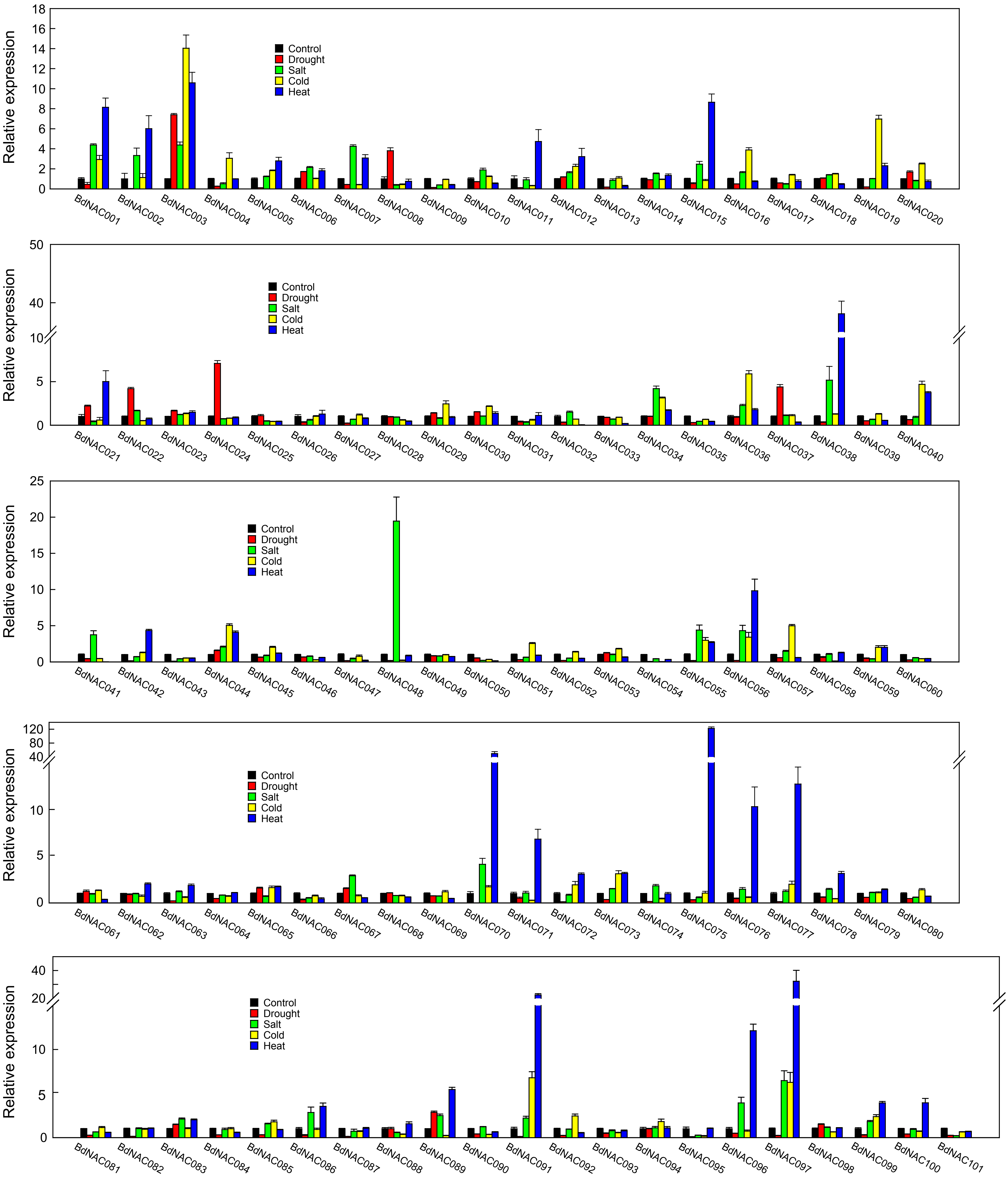

S7 Fig. Expression profile of *BdNAC* genes in response to various abiotic stresses. 3-week-old seedlings were subjected to drought stress (growth without water supply), salt (200 mM NaCl), cold (4ºC) and heat (42ºC) stresses. Relative expression levels of *BdNAC* genes were analyzed by quantitative real-time RT-PCR (qPCR). The expression levels are normalized with respect to reference gene *UBC18* in different samples. Error bars indicate standard deviations (SD) based on three technical repeats.
