# Supplementary material for: Systematic Analysis and Identification of Stress-Responsive Genes of the NAC Gene Family in Brachypodium distachyon
Source: PLoS One. 2015 Mar 27;10(3):e0122027. doi: 10.1371/journal.pone.0122027 (PMC4376915; doi:10.1371/journal.pone.0122027)
Supplement: S8 Fig — (PDF) [file pone.0122027.s008.pdf]

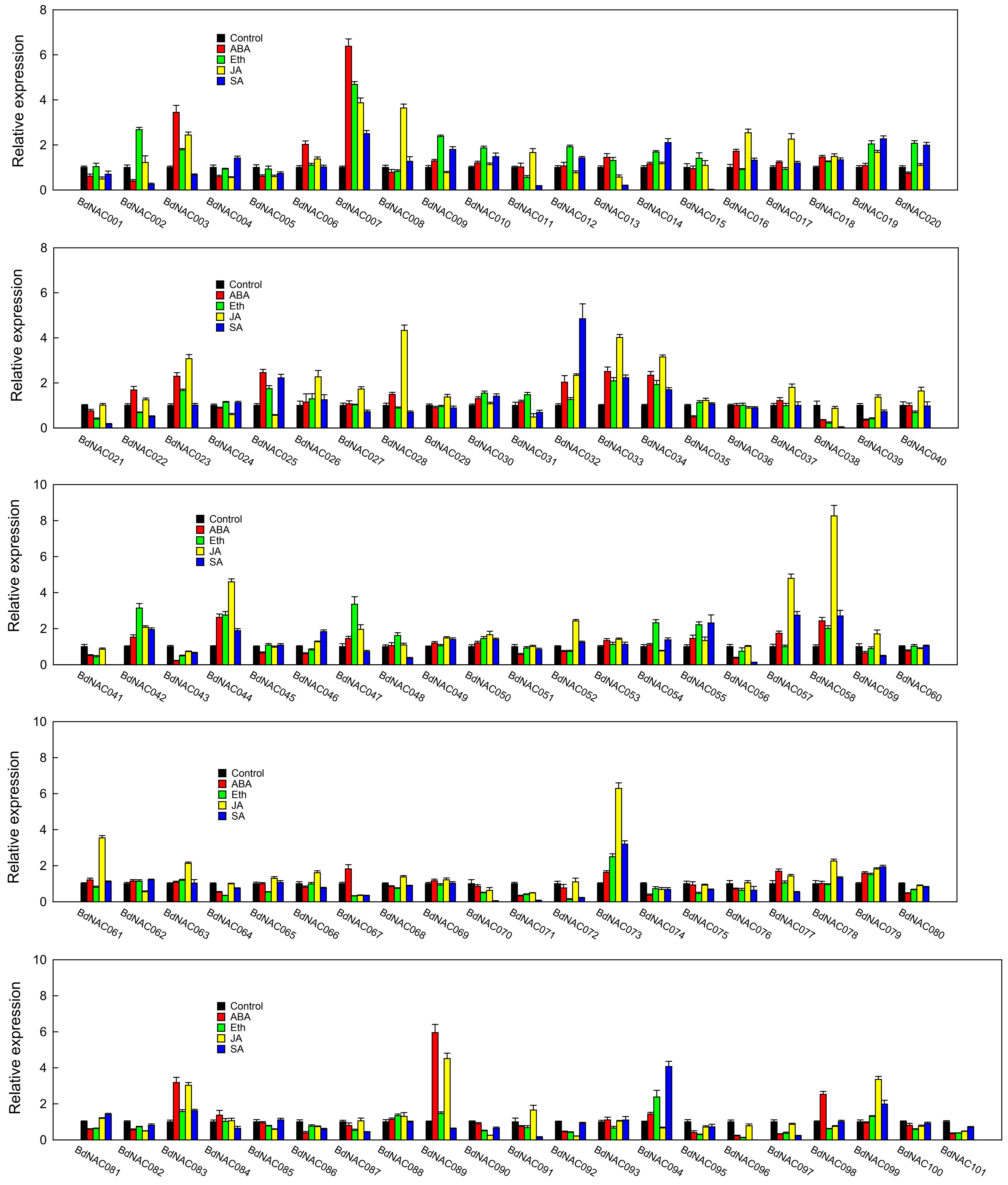

S8 Fig. Expression profile of *BdNAC* genes in response to various phytohormones. 3-week-old seedlings were treated with 100 μM ABA, 300 μM ethephon, 100 μM JA and 100 μM SA, respectively. Relative expression levels of *BdNAC* genes were analyzed by quantitative real-time RT-PCR (qPCR). The expression levels are normalized with respect to reference gene *UBC18* in different samples. Error bars indicate standard deviations (SD) based on three technical repeats.
