# Supplementary material for: Systematic Analysis and Identification of Stress-Responsive Genes of the NAC Gene Family in Brachypodium distachyon
Source: PLoS One. 2015 Mar 27;10(3):e0122027. doi: 10.1371/journal.pone.0122027 (PMC4376915; doi:10.1371/journal.pone.0122027)
Supplement: S9 Fig — (PDF) [file pone.0122027.s009.pdf]

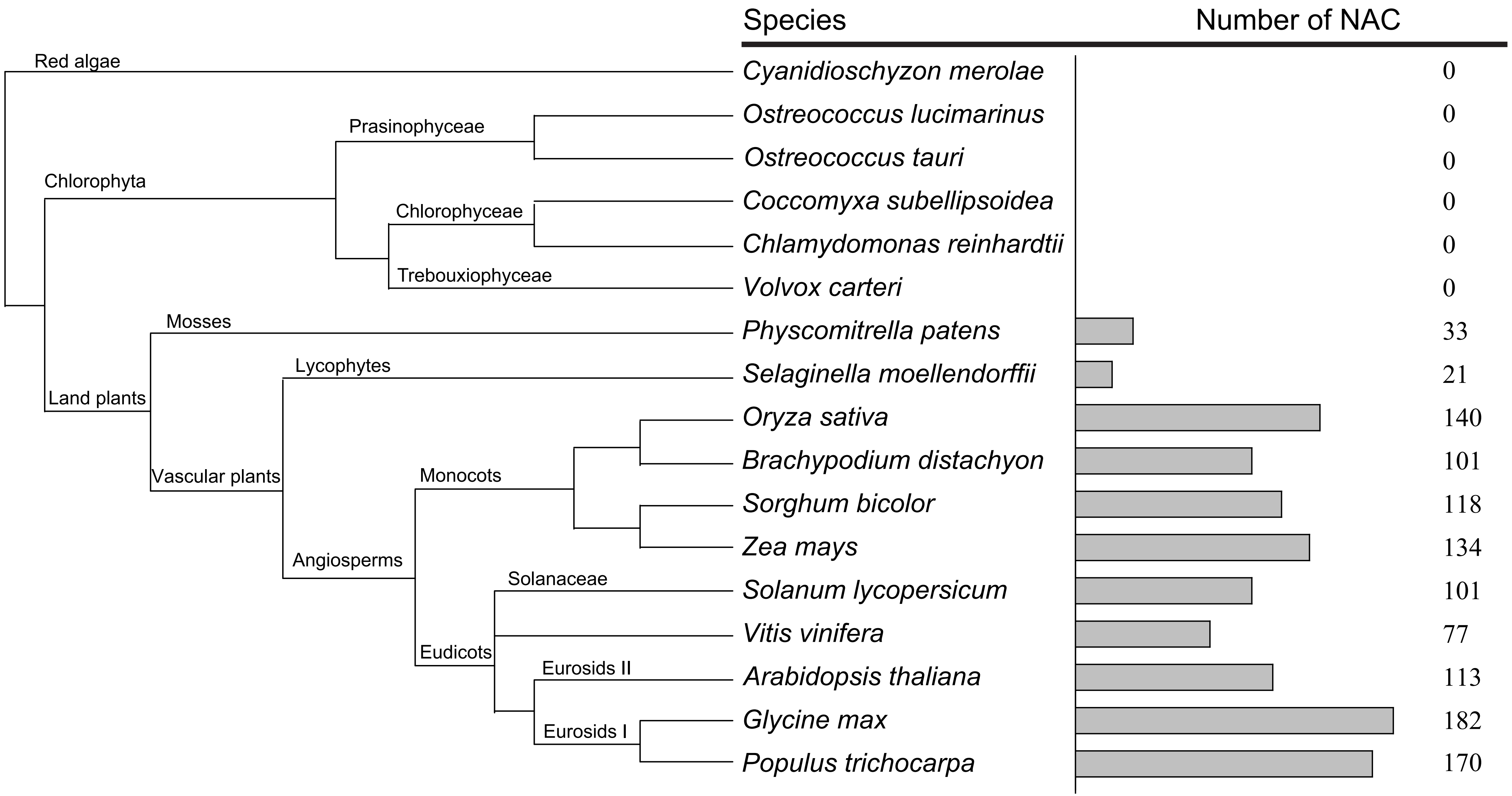

S9 Fig. Distribution of the NAC TFs in Plantae. The total number of NAC genes found in each genome is indicated on the right.
