# Supplementary material for: Systematic Analysis and Identification of Stress-Responsive Genes of the NAC Gene Family in Brachypodium distachyon
Source: PLoS One. 2015 Mar 27;10(3):e0122027. doi: 10.1371/journal.pone.0122027 (PMC4376915; doi:10.1371/journal.pone.0122027)
Supplement: S10 Fig — (PDF) [file pone.0122027.s010.pdf]

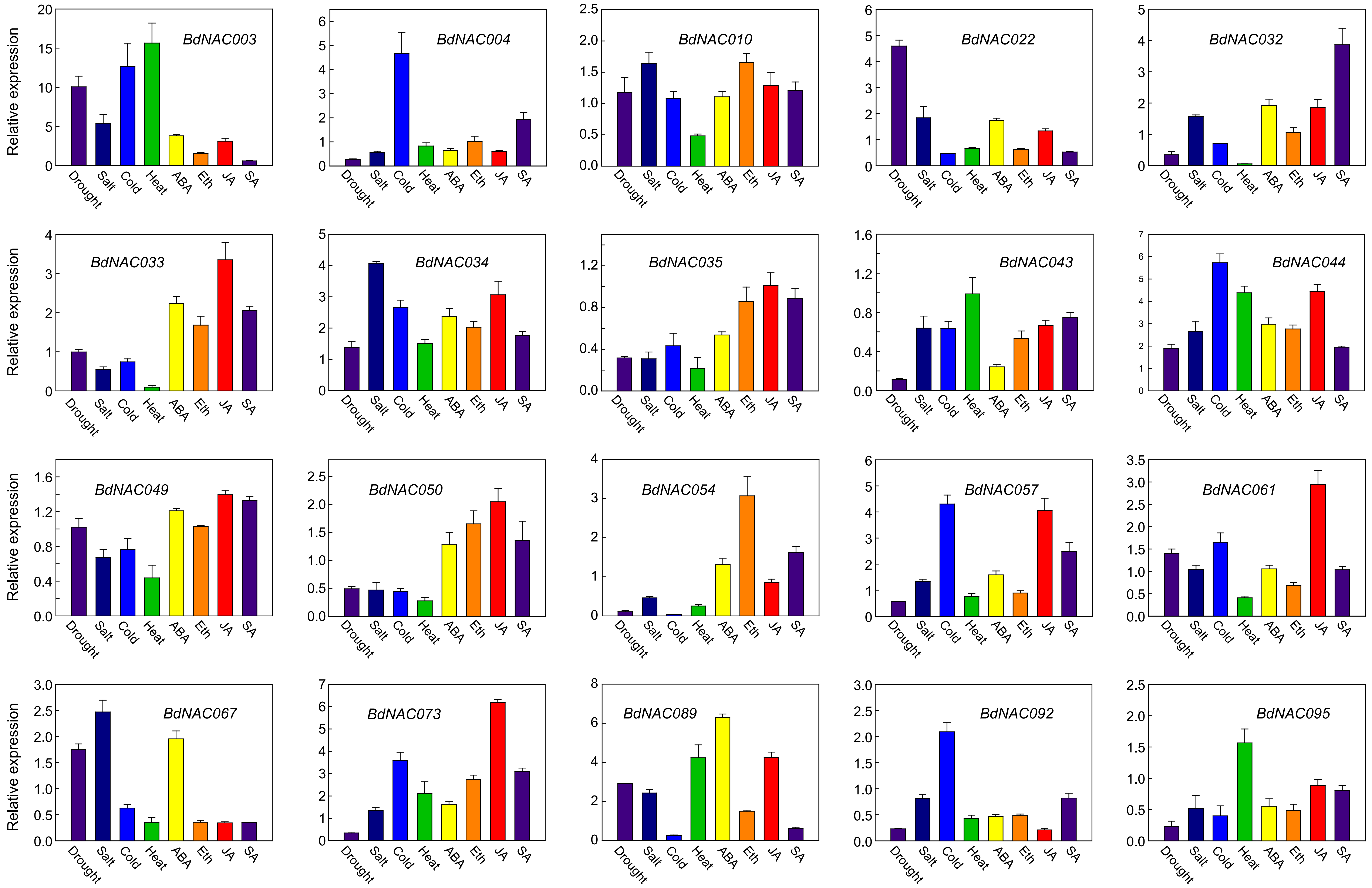

S10 Fig. Confirm the expression profiles of selected *BdNAC* genes under various abiotic stresses and phytohormone treatments by quantitative real-time RT-PCR (qPCR) with three biological repeats. The expression levels are normalized with respect to reference gene *UBC18* in different samples. The bars are standard deviations (SD) of three biological repeats.
