# Supplementary material for: Systematic Analysis and Identification of Stress-Responsive Genes of the NAC Gene Family in Brachypodium distachyon
Source: PLoS One. 2015 Mar 27;10(3):e0122027. doi: 10.1371/journal.pone.0122027 (PMC4376915; doi:10.1371/journal.pone.0122027)
Supplement: S4 Fig — (PDF) [file pone.0122027.s004.pdf]

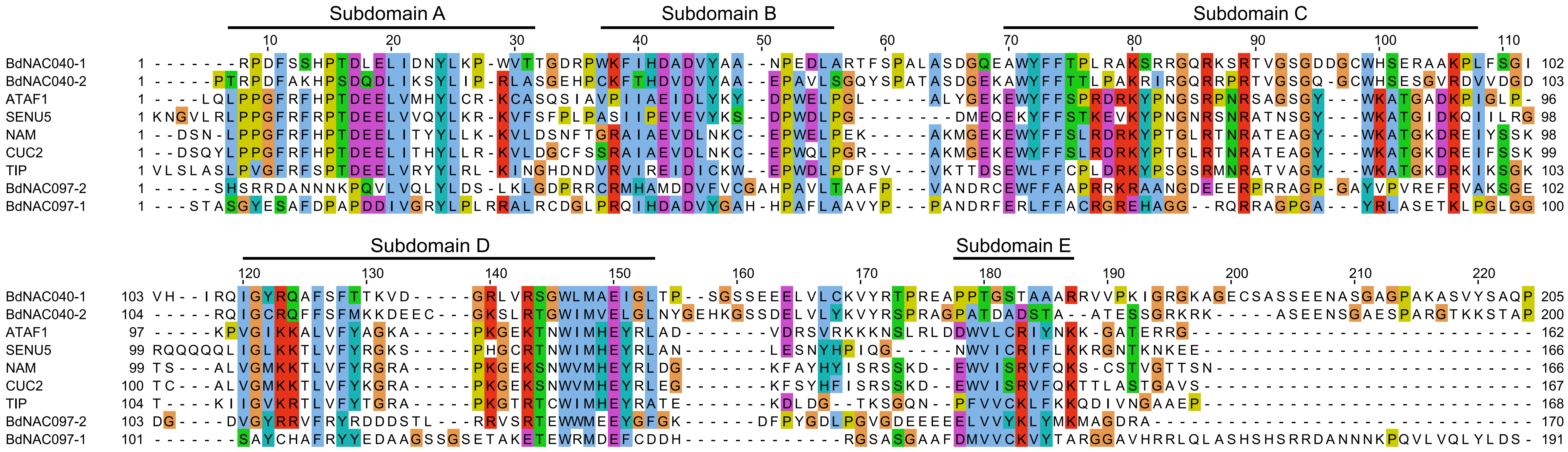

S4 Fig. Comparison of two tandem repeated NAC domain from BdNAC040 and BdNAC097. Multiple sequence alignment of NAC domain from BdNAC040, BdNAC097 and five representative Arabidopsis NACs (NAM, ATAF1, TIP, CUC2 and SENU5). The consensus NAC subdomains (A-E) are indicated by lines above the sequences.
